# Frequency of Functional Gastrointestinal Disorders and association with occupational stress among nurses at a tertiary care hospital in Karachi, Pakistan: an analytical cross-sectional study

**DOI:** 10.21203/rs.3.rs-9219713/v1

**Published:** 2026-04-01

**Authors:** Nashit Irfan Aziz, Anum Rahim, Syed Iqbal Azam, Amna Subhan Butt, Rozina Karmaliani, Asaad Ahmed Nafees

**Affiliations:** Aga Khan University; Aga Khan University; Aga Khan University; Aga Khan University; Aga Khan University; Aga Khan University

**Keywords:** Functional Gastrointestinal Disorders, Irritable Bowel Syndrome, Functional Dyspepsia, Rome IV, Occupational Stress, Nurses, Occupational Health

## Abstract

**Background:**

Functional gastrointestinal disorders (FGIDs), including irritable bowel syndrome (IBS) and functional dyspepsia (FD), are common chronic conditions characterized by persistent gastrointestinal symptoms without an identifiable organic cause. Nurses are vulnerable to occupational stress due to demanding work environments, irregular shifts, and high emotional and physical workloads, which may predispose them to FGIDs, more than general population. We assessed the frequency of FGIDs, and their association with occupational stress among nurses in Karachi, Pakistan.

**Methods:**

We conducted an analytical cross-sectional study, among 326 registered nurses at a private tertiary care hospital in Karachi, Pakistan. We applied quota sampling with proportionate allocation for recruiting participants across six major departments: Medicine, Surgery, Emergency, Outpatient, Pediatrics and Gynaecology-Obstetrics. We collected data using a self-administered questionnaire, comprising different sections: socio-demographic information, lifestyle and medical history, and the standardized Nursing Stress Scale, and Rome IV criteria for FGIDs. We reported medians with interquartile ranges for continuous variables and frequencies with percentages for categorical variables. The associations were assessed using logistic regression to calculate crude and adjusted odds ratios (aORs) with 95% confidence intervals, after checking for multicollinearity and interaction terms.

**Results:**

Frequency of FGIDs was 39% (n = 128), while approximately 20% (n = 65) experienced moderate to severe occupational stress. In the multivariable model, moderate to severe occupational stress was associated with FGIDs (aOR = 2.42, 95% CI: 1.11–5.27). Additional risk factors included history of psychiatric illness (aOR = 2.79, 95% CI: 1.46–5.32), and tobacco consumption (aOR = 3.65, 95% CI: 1.41–9.48); nurses reporting regular exercise (≥ 150 minutes/week) had 59% lower odds of FGIDs (aOR = 0.41, 95% CI: 0.23–0.71).

**Conclusions:**

We found a high burden of occupational stress and FGIDs among nurses at a tertiary care hospital in Karachi, Pakistan and found that occupational stress significantly increases the risk of FGIDs in nurses. At the institutional level, integrating screening for FGIDs into routine occupational health check-ups, alongside stress assessments, could facilitate early identification of at-risk staff.

## Background

Functional gastrointestinal disorders (FGIDs) are characterized by persistent gastrointestinal (GI) symptoms that have no apparent organic etiology. The Rome IV classification system divides these into a wide spectrum of disorders based on chronic presence of GI symptoms, with distinct cutoff durations and frequency thresholds (1, 2). Among FGIDs, two most prevalent subtypes include (a) irritable bowel syndrome (IBS), characterized by abdominal discomfort, altered bowel habits, and bloating, and (b) functional dyspepsia (FD), which presents as epigastric pain or discomfort often related to meals and associated with satiety (2). These disorders are driven by various physiological changes, including disrupted gastrointestinal motility (either accelerated or delayed), visceral hypersensitivity, imbalances in gut microbiota, and altered processing of sensory signals by the central nervous system (3). FGIDs are amongst some of the most common types of enteric health problems (4).

Global prevalence data on FGIDs are limited. A large multicentric study, conducted in the general population across 33 countries reported an overall prevalence of around 40%, with wide variation across different low-and middle-income countries (LMICs): 40% in Bangladesh, 19% in Indonesia, and only 7% in India (5). In Ethiopia, the estimated prevalence was reported as 18% for FD and 15% for IBS (6). Large variation in these estimates might indicate underlying genetic differences, and differences in culture, lifestyle, and dietary habits between countries.

Being multifactorial in nature, one of the risk factors of FGIDs is stress. However, prior studies that have investigated the link between stress and the development or severity of IBS-like symptoms, mostly concentrated on early-life (2, 7), or present-day stressors (8–11). An important source of stress, stemming from occupation, has received less attention. This can lead to changes in psychological and physical health (12). Additionally, workplace stressors, including high job demands and unfavourable work culture, were found to be associated with FD and IBS (13), with an increased prevalence of up to threefold among women employees (14).

Healthcare professionals, especially nurses, are particularly vulnerable to occupational stress due to the demanding nature of their work, long shifts, conflicts with coworkers and supervisors, exposure to suffering and death, and inadequate financial resources (15). Furthermore, irregular working hours, and disruption of circadian rhythms, puts this population at greater risk (16, 17). A study in Australia, found a higher prevalence of FGIDs in nurses when compared to physicians and allied health professionals, with 27% reporting FGID symptoms (18). Prolonged sources of stress not only affect mental health outcomes like anxiety, depression, and burnout, but also impact on the quality of patient care (19). Since nurses form a huge part of a hospital’s workforce, and are forefront carers for patients, identifying such factors is crucial.

In Pakistan, FGIDs remain largely understudied, as reflected by the limited availability of data. A study conducted in Karachi indicated a prevalence of about 54% in the general population (20). Several other studies that have reported FGIDs, have solely focused on IBS (21–23). No study, however, reported the prevalence of FGIDs, among Pakistani nurses. Furthermore, the potential relationship between occupational stress and FGIDs warrants further enquiry; and if it holds true, implementing workforce interventions could address a preventable risk factor of FGIDs. Against this background, our study aimed to estimate the frequency of FGIDs and to determine association between occupational stress and FGIDs among nurses working at a tertiary care hospital in Karachi, Pakistan.

## Methods

### Study design, study setting and sample

We conducted an analytical cross-sectional study between July and September 2025 at a private tertiary care hospital in Karachi, Pakistan. The sample size was estimated using OpenEpi (version 3.0) with a 95% confidence level and 80% power. For estimating prevalence, prior studies reported FGIDs among nurses ranging from 27–29% (Australia, India) (18, 24), which yielded a minimum sample size of 242 participants. For the association between occupational stress and FGIDs, assuming an odds ratio of 2.0 (25), 50% exposure prevalence, and equal ratio of exposed/unexposed (20, 26), the required sample size was 296. After accounting for a 10% non-response rate, the final target was 326. A non-probability quota sampling method was employed to recruit participants, with proportionate allocation across six major hospital departments, where the collective strength of nurses was 842.

### Eligibility criteria

We included all registered nurses (RNs) who had been working for at least a year. Nurses who were pregnant, and those not directly involved in clinical care such as head nurses, supervisors, coordinators, or nurse managers, were excluded.

### Data collection

Data were collected using a structured self-administered questionnaire comprising of four sections by a trained team. Section A captured sociodemographic and job-related characteristics, section B captured occupational stress among nurses, section C included questions of medical history and lifestyle factors and section D included assessment of FGIDs, as per Rome IV criteria.

Data were gathered electronically using tablets (via Epicollect) in person by research assistants who received standardized training on participant approaches, ethical conduct, and data entry. Participants were asked to recall exposure during the last six months. Each participant was assigned a unique code to prevent duplicate responses. Data collection proceeded department-wise in a phased approach to minimize workflow disruption and in a non-clinical area in each ward to ensure privacy. The study questionnaire was piloted on 18 nurses (5% sample) prior to initiation of data collection and minor changes in flow and phrasing of questions were incorporated. The data were monitored in real-time for completeness and accuracy.

### Data Collection Instruments

Occupational stress was measured using the standardized Nursing Stress Scale (NSS) (27), a 34-item instrument with seven subscales, including: *Death and Dying, Interpersonal conflict, Inadequate preparation, Lack of support, Workplace conflict and safety, Workload, and Uncertainty concerning treatment*. This tool has been previously validated in LMICs, with strong internal dependability, a Cronbach’s alpha of 0.89, and a test-retest coefficient of 0.81 (27). All items within these domains were rated on a 4-point scale: Never, Occasionally, Frequently, Very Frequently. The n (%) values indicate the number and percentage of nurses who selected each option for each item in the questionnaire.

FGIDs was diagnosed using the gold-standard criteria (Rome IV criteria) (2), to reduce measurement bias. FGIDs were limited to Functional Dyspepsia (FD) and Irritable Bowel Syndrome (IBS) in our study, and participants were classified as FGID-positive if they met the diagnostic thresholds for either condition or both.

Covariates included age, sex, marital status, having children, living setup, duration of employment, rotating shift, average sleep duration, average exercise duration, past medical history, tobacco and alcohol consumption.

### Statistical analysis

Data analysis was performed using STATA 17.0. All descriptive statistics were reported as frequencies and percentages for categorical variables and medians with interquartile ranges for the skewed continuous variables. Univariate analysis was used to identify the predictors of FGIDs (p ≤ 0.25). Variables meeting this threshold were entered into a multivariable logistic regression model after checking for multicollinearity using r > 0.8 as cut-off. Adjusted odds ratios (aOR) and confidence intervals (CIs) were reported at p ≤ 0.05.

## Results

**Figure 1:**
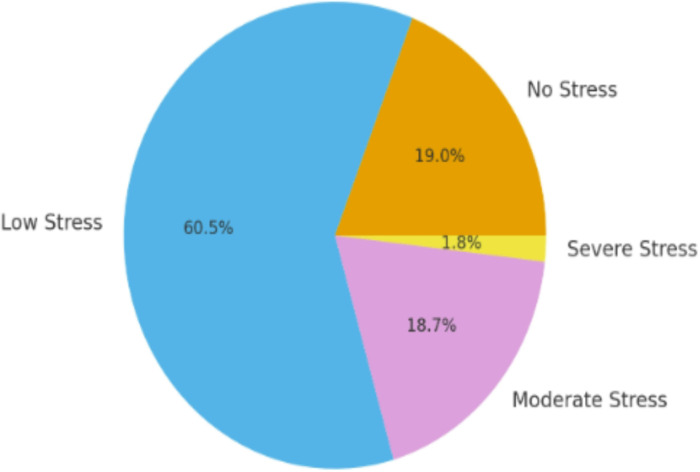
Frequency of occupational stress among nurses(n= 326)

Among 326 nurses, the median age was 27 (25–30) years, with a predominance of females in the sample (approximately 60%). Furthermore, occupational factors, social support factors, past medical history and lifestyle factors were also taken into consideration (Table 1 and Fig. 1).

Stressors factors related to *Death and Dying* and *Workload* were the most reported domains, with about one-third of nurses indicating they encountered these situations “Frequently” or “Very Frequently.” These domains encompassed emotionally taxing events such as caring for terminally ill patients, witnessing suffering, and managing excessive workload demands. In contrast, stressors associated with *Workplace Conflict* and *Safety and Lack of Support* were reported less often, suggesting relatively fewer interpersonal or organizational difficulties in our study setting. The detailed item-wise distribution of responses for each domain is provided in Table 2, offering a comprehensive view of specific stressors within each category.

Out of 326 nurses, 128 (39%) were identified as having FGIDs. Functional dyspepsia was the predominant subtype, affecting 128 (39.3%) participants, whereas irritable bowel syndrome was relatively uncommon (11, 3.4%). Notably, all IBS cases co-occurred with functional dyspepsia.

Univariate analysis was conducted on 326 participants using logistic regression to examine the relationship between various factors and the presence or absence of FGIDs. The results are summarized in Table 3. After running the univariate analysis, the correlation between each independent variable was checked to limit the chances of multicollinearity. Pearson’s correlation assessed quantitative variables, Cramer V assessed qualitative variables, and Eta assessed combination of qualitative and quantitative variables. Multicollinearity was found between age and total nursing experience (r = 0.87); therefore, age was dropped from the final model. Using backward and forward selection, variables from the univariate analysis were entered for multivariable model building, where p ≤ 0.05 was significant. In our sample, no confounding or interaction was significant (p > 0.1).

**Figure 2:**
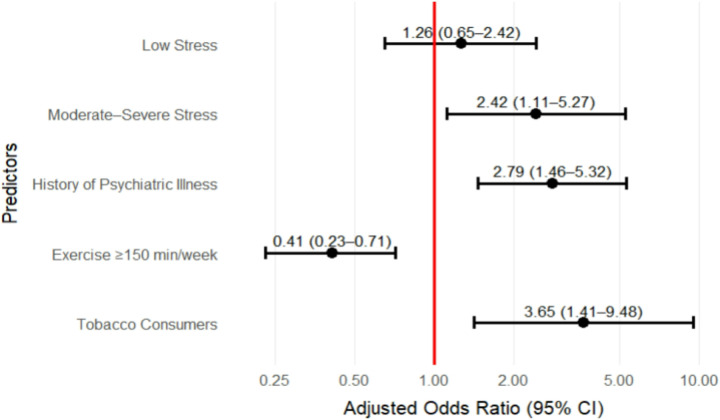
Predictors of FGIDs among nurses (n = 326) in final regression model

In the final model, several factors remained strongly associated with FGIDs among nurses. Nurses experiencing moderate to severe work-related stress had more than twice the likelihood of developing FGIDs compared to those who reported no stress (aOR = 2.42, 95% CI: 1.11–5.27). A history of psychiatric illness emerged as a significant predictor, with affected nurses having nearly three times the odds of FGIDs compared to those without such a history (aOR = 2.79, 95% CI: 1.46–5.32). Likewise, tobacco consumption maintained its significant association in the adjusted model, with individuals who had ever used tobacco showing over three times the odds of FGIDs compared to those who had never used it (aOR = 3.65, 95% CI: 1.41–9.48). In contrast, regular physical activity appeared to offer protection. Nurses who engaged in at least 150 minutes of exercise per week had significantly lower odds of FGIDs compared to those who exercised less often (aOR = 0.41, 95% CI: 0.23–0.71). The forest plot in Fig. 2. shows the final model.

Our model demonstrated a satisfactory goodness of fit (Pearson χ^2^ = 11.78, p = 0.55), confirming the robustness of the estimates.

## Discussion

We found a high frequency of FGIDs (39%) among Pakistani nurses; these were associated with occupational stress, history of psychiatric illness, and tobacco consumption. Weekly average exercise duration ≥ 150 minutes was found to be a protective factor for FGIDs.

Although nationwide data on FGIDs in Pakistan are lacking, a Karachi-based study estimated a 54% overall prevalence in the general population, with 9% for IBS and 38% for FD (20). In comparison, our study found an 11% prevalence of IBS, a slightly higher percentage, which might be attributed to other occupational factors such as irregular sleep patterns and circadian rhythm disturbances, which are common among nurses. Additionally, FD was more prevalent than IBS within our study. Differences between our study and international studies, such as the 27% prevalence of FGIDs among Australian nurses (18), may stem from different sociodemographic or working environments. Additionally, all IBS-positive cases in our study had FD, suggesting that nurses may more readily recognize and report overlapping gastrointestinal symptoms that may go unnoticed in the general population.

Nurses experiencing moderate to severe occupational stress (20%) had twice the odds of developing FGIDs (aOR = 2.42, 95% CI: 1.11–5.27), when adjusted for other factors. To date, evidence investigating the link between these variables among different occupational groups is scarce and even more so among nurses. The only study among nurses investigating the impact of occupational stressors on FGIDs was conducted in 2003 at a Dutch university hospital, where the authors concluded that having lower task control leads to 32% higher odds for IBS, compared to those with higher task control (28).

We found that nurses with a history of psychiatric illness were nearly three times more likely to report FGIDs (aOR = 2.79, 95% CI: 1.46–5.32). Psychiatric disorders, particularly anxiety and depression, are highly comorbid with FGIDs. This strong association is consistent with literature. A meta-analysis from China reported 2.35 times higher odds of developing IBS among university students who had anxiety, and 2.15 times higher odds of developing IBS in those who had depression (29). This reinforces the critical role of the gut-brain axis in the pathophysiology of FGID. A cross-sectional study among medical students in Khyber Pakhtunkhwa province, Pakistan, reported significant associations between depression and anxiety and FGIDs (30). Psychological distress likely worsens symptom severity, impairs quality of life, and reduces work performance - issues that are directly relevant to nursing efficiency and patient care (31, 32).

The finding that individuals who had ever used tobacco exhibited over three times the odds of developing FGIDs represents a plausible argument. Although some studies have presented contradictory evidence or weak associations between tobacco use and FGIDs, the broader literature on gastrointestinal health offers a biological basis for this association. For instance, a large meta-analysis (n = 2560) based on three population-based studies in Sweden concluded that consuming 11–20 cigarettes per day increased the odds of FD by 42%, while consuming more than 20 cigarettes per day doubles the odds. The same study found association between IBS (diarrhea subtype) and smoking, with an odds ratio (OR) of 2.40, although there was no association with other subtypes of IBS (33). Though our study corroborated with this meta-analysis, the strength of the association should be interpreted with caution, primarily because in our study tobacco was not our primary variable and so it was not defined in terms of its intensity, type, duration, and frequency. Interestingly, a study from Karachi, Pakistan, also concluded that non-smokers had three fold higher odds of IBS compared to smokers (34), which might be attributed to selection bias or uncontrolled confounding lifestyle factors. This suggests that further studies in Pakistan are needed to elucidate the associations between tobacco consumption and FGIDs. Nonetheless, smoking cessation remains an advisable intervention, based on previous research and the current study.

Our study identified a protective effect of physical activity for nurses engaging in ≥ 150 minutes of weekly exercise (aOR = 0.41, 95% CI: 0.23–0.71) which is consistent with previous studies. A randomized controlled trial (RCT) offered the most compelling support for this relationship, where IBS patients in a structured physical activity program showed a much larger decrease in symptom severity than the control group, with a mean decrease in the IBS Severity Scoring System of 51 points in the intervention arm compared with 5 points in the control arm (p = 0.003). A recent Cochrane meta-analysis, which reviewed six RCTs (n = 185), also found that participating in physical activity resulted in an improvement in overall IBS symptoms (35). Our study’s findings for physical activity’s protective effect on FGIDs, is in line with other regional evidence. A large population-based study from Iran with 4,763 adults reported that individuals with < 1 hour/week of activity had 1.27 times higher odds of IBS (95% CI: 1.08–1.49), when compared to more active participants (36). The results highlight the importance of physical activity as a modifiable behaviour that affects the risk of FGID in different population groups Physiologically, exercise improves gut motility, reduces gut inflammation, protects the gut barrier (37), and positively affects the gut–brain axis by reducing stress, anxiety, and depression (38).

Our study had a few strengths. For instance, we used validated tools for measuring FGIDs and occupational stress. Secondly, our sampling technique ensured that we had a representative sample of nurses from the hospital. Thirdly, the data were collected by a co-investigator and their team members who received field training about the study using a manual of operations. This manual contained various steps to limit problems during questionnaires administration and immediate verification of responses during data collection phase, thereby reducing measurement errors.

### Limitations

Since cross sectional studies are prone to common-method bias, they are unable to establish causality between occupational stress and FGIDs, which was a design limitation in our study. Future studies could consider case control or cohort studies to establish causality. The results of the study were based on a private tertiary care hospital, which in Pakistan typically have better staffing ratios and fewer workplace violence incidents than public hospitals, therefore our findings might be under-representing the burden. However, our decision was based on logistical constraints for including a single center. Multi-center studies involving both the public and private sectors could be another direction for future research. A potential healthy worker bias may also have led to an underestimation of the burden of occupational stress and FGIDs, as those who were very sick may have already left employment, while those relatively healthier remained in the workforce. The use of self-administered questionnaires introduces the potential for under- or over-reporting occupational stress, anxiety/depression and FGID symptoms. No clinical assessments were performed to verify FGID diagnoses; therefore, the prevalence estimates, though based on standard criteria, may not reflect true clinical prevalence. Lastly, there is a potential for residual confounding in the study from unmeasured factors such as diet, BMI, sleep quality, or personality traits. However, their inclusion would have increased the time duration and potentially reduced response accuracy for the main exposure. Future studies could consider multi-domain data collection integrating more lifestyle and biological parameters or different occupational populations to refine the explanatory model.

## Conclusion

Our research found a high burden of functional gastrointestinal disorders among nurses, with occupational stress emerging as an important contributing factor. Additionally, past history of psychiatric illness and smoking were strongly associated with the occurrence of FGIDs; weekly exercise ≥ 150 minutes was a protective factor. Our findings highlight the multifactorial nature of these conditions and emphasize the importance of using a biopsychosocial approach when studying FGIDs in nursing populations. Future studies may explore stress-related and behavioral influences in healthcare settings and preventive strategies.

## Figures and Tables

**Table 1:** Summary table of socio-demographic and lifestyle characteristics among nurses (n = 326)

Variables	Categories/Median (IQR)	n (%)
Age; in years^+^	27 (25–30)	-
Gender	Females	190 (58.28)
	Males	136 (41.72)
Marital status	Single	207 (63.50)
	Married	118 (36.20)
	Divorced	01 (0.31)
Children	Yes	82 (25.00)
	No	244 (75.00)
Nursing education	Diploma in Nursing	60 (19.40)
	BScN	266 (81.60)
Living setup	Alone	49 (15.03)
	With family/friends	277 (85.00)
Total nursing experience; in years^+^	3 (2–6)	-
Experience at current center; in years^+^	2 (1–5)	-
Hospital department	Medicine	95 (29.14)
	Surgery	50 (15.34)
	ED	51 (15.64)
	OPD	20 (6.13)
	Gynae-Obs	20 (6.13)
	Paediatrics	90 (28.00)
Rotating shift	Yes	297 (91.10)
	No	29 (9.00)
History of psychiatric illness*	None	274 (84.10)
	Present	52 (15.90)
History of GI diseases**	None	237 (72.70)
	Present	89 (27.30)
History of other chronic conditions***	None	283 (86.80)
	Present	43 (13.20)
Tobacco consumption^	Never	304 (93.30)
	Current	12 (4.00)
	Previous	10 (3.10)
Alcohol consumption	Non-consumer	321 (99.00)
	Consumer	5 (2.00)
Exercise duration per week; in minutes	< 150	229 (70.25)
	≥ 150	97 (29.75)
Sleep duration per night; in hours	< 7	185 (56.75)
	≥ 7	141 (43.25)

* History of Psychiatric illness includes anxiety and depression.

** History of GI diseases include esophageal, gastric and intestinal diseases

*** History of other chronic conditions include hypertension, diabetes, asthma, thyroid disorders

+ Medians/IQR for continuous variables (Skewed)

^ Tobacco consumed in any form.

**Table 2:** Frequency distribution of responses for Nursing Stress Scale (n = 326)

Domains	Never n (%)	Occasionally n (%)	Frequently n (%)	Very frequently n (%)
1. Death and Dying (7 items)				
Performing procedures that patients experience as painful	103 (32.00)	142 (44.00)	66 (21.00)	15 (5.00)
Feeling helpless in the case of a patient who fails to improve	9 (3.00)	105 (32.21)	137 (42.02)	75 (23.01)
Listening or talking to a patient about his/her approaching death	69 (21.20)	125 (38.34)	89 (27.30)	43 (13.20)
The death of a patient	61 (19.00)	198 (61.00)	50 (15.34)	17 (5.21)
The death of a patient with whom you developed a close relationship	94 (29.00)	184 (56.44)	37 (11.40)	11 (3.40)
Physician not being present when a patient dies	35 (11.00)	135 (41.41)	108 (33.13)	48 (15.00)
Watching a patient suffer	25 (8.00)	99 (30.40)	110 (34.00)	92 (28.22)
2. Interpersonal Conflict (5 items)				
Criticism by a physician	122 (37.42)	156 (48.00)	40 (12.30)	8 (2.50)
Conflict with a physician	42 (13.00)	164 (50.31)	77 (24.00)	43 (13.20)
Fear of making a mistake in treating a patient	114 (35.00)	172 (53.00)	32 (10.00)	8 (3.00)
Disagreement concerning the treatment of a patient	133 (41.00)	130 (40.00)	46 (14.11)	17 (5.21)
Making a decision concerning a patient when the physician is unavailable	64 (20.00)	158 (49.00)	71 (22.00)	33 (10.12)
3. Inadequate Preparation (3 items)				
Feeling inadequately prepared to help with the emotional needs of a patient’s family	164 (50.31)	122 (37.42)	27 (8.30)	13 (4.00)
Being asked a question by a patient for which I do not have a satisfactory answer	142 (44.00)	130 (40.00)	46 (14.11)	8 (3.00)
Feeling inadequately prepared to help with the emotional needs of a patient	33 (10.12)	152 (47.00)	109 (33.44)	32 (10.00)
4. Lack of Support (3 items)				
Lack of an opportunity to talk openly with other unit personnel about problems on the unit	117 (36.00)	130 (40.00)	59 (18.10)	20 (6.13)
Lack of an opportunity to share experiences and feelings with other personnel on the unit	82 (25.20)	112 (34.40)	84 (26.00)	48 (15.00)
Lack of an opportunity to express to other personnel on the unit my negative feelings toward patients	127 (39.00)	163 (50.00)	31 (10.00)	5 (2.00)
5. Workplace Conflict & Safety (5 items)				
Conflict with a supervisor	163 (50.00)	133 (41.00)	25 (8.00)	5 (2.00)
Floating to other units that are short-staffed	241 (74.00)	62 (19.02)	19 (6.00)	4 (1.23)
Difficulty in working with a particular nurse (or nurses) outside the unit	136 (42.00)	137 (42.02)	39 (12.00)	14 (4.30)
Criticism by a supervisor	150 (46.01)	131 (40.20)	35 (11.00)	10 (3.10)
Difficulty in working with a particular nurse (or nurses) on the unit	89 (27.30)	161 (49.40)	61 (19.00)	15 (5.00)
6. Workload (6 items)				
Breakdown of computer	12 (4.00)	106 (33.00)	144 (44.20)	64 (20.00)
Unpredictable staffing and scheduling	45 (14.00)	155 (48.00)	83 (26.00)	43 (13.20)
Too many non-nursing tasks required, such as clerical work	20 (6.13)	134 (41.10)	100 (31.00)	72 (22.10)
Not enough time to provide emotional support to a patient	59 (18.10)	160 (49.10)	73 (22.40)	34 (10.43)
Not enough time to complete all of my nursing tasks	20 (6.13)	124 (38.04)	97 (30.00)	85 (26.10)
Not enough staff to adequately cover the unit	75 (23.01)	145 (45.00)	62 (19.02)	44 (14.00)
7. Uncertainty Concerning Treatment (5 items)				
Inadequate information from a physician regarding the medical condition of a patient	44 (14.00)	129 (40.00)	98 (30.10)	55 (17.00)
A physician ordering what appears to be inappropriate treatment for a patient	26 (8.00)	146 (45.00)	102 (31.30)	52 (16.00)
A physician not being present in a medical emergency	87 (27.00)	115 (35.30)	83 (26.00)	41 (13.00)
Not knowing what a patient or a patient’s family ought to be told about the patient’s condition and its treatment	89 (27.30)	159 (49.00)	59 (18.10)	19 (6.00)
Uncertainty regarding the operation and functioning of specialized equipment	41 (13.00)	123 (38.00)	94 (29.00)	68 (21.00)

**Table 3:** Univariate Analysis for factors associated with FGIDs among nurses

Study Variables	Unadjusted OR (95% CI)
Occupational stress* (ref = No Stress)	
Low Stress	1.56 (0.83–2.92)
Moderate-Severe Stress	3.47 (1.66–7.26)
History of psychiatric illness* (ref= None)	
Present	2.97 (1.61–5.47)
Exercise duration per week; in minutes* (ref < 150)	
≥ 150	0.43 (0.25–0.72)
History of GI diseases* (ref = None)	
Present	2.30 (1.40–3.77)
Tobacco consumption* (ref=Never consumers)	
Ever consumers	2.92 (1.19–7.17)
Sleep duration per night; in hours* (ref = < 7)	
≥ 7	0.58 (0.36–0.91)
Living setup* (ref=Alone)	
Family/Friends	0.52 (0.28–0.95)
Age*	
every 5 year increase	0.83 (0.68–1.00)
Rotating shifts* (ref = No)	
Yes	2.16 (0.89–5.22)
Gender* (ref=Female)	
Male	0.67 (0.43–1.06)
Marital status* (ref=Single)	
Married	0.69 (0.43–1.11)
Total job experience*	
every 10 years	0.73 (0.46–1.16)
Children (ref = No)	
Yes	0.75 (0.44–1.26)
Nursing education (ref=Bachelor’s Degree in Nursing)	
Diploma in Nursing	0.73 (0.41–1.32)
Alcohol consumption (ref = non-consumers)	
Consumers	2.35 (0.39–14.27)
Hospital departments (ref=Medicine & Allied)	
Surgery and Allied	0.91 (0.45–1.88)
Emergency Department	1.70 (0.86–3.39)
Outpatient Department	0.70 (0.25–1.99)
Gynae-Obs	0.70 (0.25–1.99)
Paediatrics	1.09 (0.60–1.97)
Duration of employment at current center	
every 5 years	0.94 (0.73–1.22)
History of other chronic diseases (ref = None)	
Present	1.01 (0.53–1.95)

* Statistically significant, p ≤ 0.25

## Data Availability

The datasets utilized during this study are available from the corresponding author on reasonable request.

## Abbreviations

GI: Gastrointestinal
FGIDs: Functional Gastrointestinal Disorders
IBS: Irritable Bowel Syndrome
FD: Functional Dyspepsia
LMICs: Low-and Middle-Income Countries
NSS: Nursing Stress Scale
RN: Registered Nurses
OPD: Outpatient Departments
OR: Odds Ratio
CI: Confidence Interval
